# Relationship between socioeconomic and nutritional status in the Serbian adult population: a cross-sectional study

**DOI:** 10.1590/1516-3180.2018.0038170418

**Published:** 2018-08-13

**Authors:** Jelena Gudelj Rakić, Miloš Maksimović, Janko Janković, Hristina Vlajinac, Jelena Marinković

**Affiliations:** I MD, PhD. Medical Doctor, Institute of Public Health of Serbia “Dr Milan Jovanović Batut”, Belgrade, Serbia.; II MD. Professor, Institute of Hygiene and Medical Ecology, Faculty of Medicine, University of Belgrade, Belgrade, Serbia.; III MD. Associate Professor, Institute of Social Medicine, Faculty of Medicine, University of Belgrade, Belgrade, Serbia.; IV MD. Professor, Institute of Epidemiology, Faculty of Medicine, University of Belgrade, Belgrade, Serbia.; V PhD. Statistician and Professor, Institute of Medical Statistics and Informatics, Faculty of Medicine, University of Belgrade, Belgrade, Serbia.

**Keywords:** Socioeconomic factors, Obesity, Cross-sectional studies, Health surveys, Serbia

## Abstract

**BACKGROUND:**

Socioeconomic status is a well-known risk factor for obesity. The aim of this study was to assess the relationship between socioeconomic and nutritional status in the Serbian adult population.

**DESIGN AND SETTING:**

Cross-sectional study on data from the 2013 National Health Survey performed in Serbia.

**METHODS:**

The study population consisted of adults aged ≥ 20 years. Face-to-face interviews and anthropometric measurements were conducted by trained staff. Associations between body mass index and sociodemographic variables were analyzed using multivariable logistic regression analyses.

**RESULTS:**

Out of 12,461 subjects of both sexes, 36.4% were overweight and 22.4% were obese. The prevalences of overweight and obesity differed significantly between the sexes, regarding all sociodemographic characteristics. Among women, educational attainment was associated with lower risk of being overweight (odds ratio, OR = 0.82; 95% confidence interval, CI: 0.69-0.98 for medium-level and OR = 0.77; CI: 0.62-0.97 for higher education) or obese (OR = 0.68; CI: 0.57-0.82 for medium-level and OR = 0.41; CI: 0.31-0.54 for higher education). In contrast, medium-level (OR = 1.28; CI: 1.08-1.52) and highly educated men (OR = 1.39; CI: 1.11-1.74) were more frequently overweight than were those with low education. Among men, grade I obesity was positively related to the richest wealth index group (OR = 1.27), while the opposite was true for grade II obesity among women (OR = 0.61).

**CONCLUSION:**

This study showed significant socioeconomic inequalities in nutritional status between men and women. Continuous monitoring of socioeconomic patterns relating to weight is important, especially with further exploration of the link between education and obesity.

## INTRODUCTION

Overweight and obesity are a growing public health problem worldwide. The prevalence is increasing rapidly in countries of all income levels (high, medium and low). Since 1975, obesity levels have nearly tripled worldwide. In 2016, 39% of adults aged 18 years and over were overweight and 13% were obese.^1^ In Serbia, as globally, the increasing prevalence of overweight and obesity is an important public health challenge. In 2013, among adults aged 20 years and over, 56.3% were overweight and 21.2% were obese,^2^ whereas in 2006 these figures were 54.5% and 18.3%, respectively.^3^ The rise in obesity in Serbia, as in other middle-income countries, is likely due to a variety of factors, including increased food energy supply, economic transition, globalization of the world food market, and social and cultural changes.^4^ However, there is currently a lack of detailed analysis of obesity in Serbia.

Socioeconomic status has been found to be an important factor associated with obesity. In most studies, occupation, education and income are used as socioeconomic status indicators and the results regarding their associations with obesity vary depending on the type of study, population selected, gender, age and indicators used.^5^^,^^6^ In high-income countries, an inverse relationship between socioeconomic status and obesity has been reported,^7^^,^^8^ while in middle and low-income countries, studies have shown a strong direct relationship between socioeconomic status and overweight/obesity both among men and among women.^9^^,^^10^^,^^11^^,^^12^

The objective of the present study was to assess the relationship between socioeconomic and nutritional status in the Serbian adult population.

## METHODS

### Setting, study design and ethical concerns 

We used cross-sectional data on the Serbian adult population from the 2013 National Health Survey, which has been described in detail elsewhere.^13^ The Ethics Review Board of the Institute of Public Health of Serbia (Decision number 5996/1, of October 1, 2013) and the Ministry of Health of the Republic of Serbia issued the necessary approval for undertaking this study.

### Study population

The sample was chosen by using a stratified, two-stage nationally representative random sampling approach, using population data from census records that were generated in the year 2011. Four geographical regions of Serbia, including urban and rural areas (Vojvodina, Belgrade, Šumadija and Western Serbia, and Eastern and Southern Serbia) were identified in the sample. The units of the first stage of sampling were census enumeration areas, while the units of the second stage of sampling were randomly selected households.

A total of 670 census enumeration areas within each region with probability proportional to size were selected during the first stage. Households were selected using computer-generated simple random sampling without replacement. The number of households selected in each selected enumeration area was 10, plus three backup households. Representatives from backup households were interviewed only if individuals in some of the first 10 households were not found. If the members of a household refused to be interviewed, no backup household was contacted.

In the end, out of a total of 10,089 households that were contacted, members of 6,500 households agreed to participate in the survey, thus constituting a response rate of 64.4%, and personal visits were made to each one of these households. Out of the 12,722 members of these households who were aged 20 years and over, 12,461 were interviewed, which yielded a response rate of 97.9%. Face-to-face interviews and anthropometric measurements were carried out at participants’ homes by trained staff, consisting of two interviewers and a healthcare worker. All respondents were informed about the purpose of the study and gave their written consent to participate.

### Study variables

The variables assessed included sociodemographic characteristics (age, sex, region, education, marital status, employment status, wealth index, as calculated below, and type of settlement) and objective findings (weight, height and waist circumference). These were used to analyze socioeconomic inequalities according to sex and nutritional status, among the subjects selected. Standard procedures for measurements of weight, height and waist circumference were used. Body mass index was categorized according to the World Health Organization criteria.^14^

The following variables were used to reflect socioeconomic position in this study: educational level, defined in three categories, i.e. low (≤ 8 years), medium (8-12 years) and high (≥ 12 years); employment status, categorized as employed, economically inactive or unemployed; and wealth index, which was used to measure household wealth and thus categorized the respondents into three socioeconomic groups, i.e. low, medium and high on the basis of their assets. Generally, all items that could give a picture of socioeconomic position were used as variables in the wealth index calculation. These items included the number of bedrooms per household member; main material used for the floor, roof and walls of the house; main source of drinking water; means of sanitation; energy source used for heating; and possession of color television, mobile phone, refrigerator, computer, washing machine, dishwasher, air conditioning, central heating, car and internet access. Factor analysis and principal component analysis (PCA) were used to assign weights or factor scores to each variable.^15^ Details about how the wealth index was calculated and other variables were determined have been provided elsewhere.^13^^,^^15^^,^^16^

### Statistical analysis

Continuous variables were described in terms of means and standard deviations, while categorical variables were expressed as frequencies and percentages. Prevalence rates with appropriate 95% confidence intervals (CI) were estimated for the six categories of body mass index (BMI), separately for males and females. All reported age-adjusted estimates were weighted using probability-sampling weights. The impact on the precision of stratification of the sampling weights from the variance estimates and confidence intervals reported was accounted for by using Taylor-series linearization techniques for complex samples. The chi-square test, Student’s t test, Mann-Whitney U test, Kruskal-Wallis test and one-way analysis of variance with the post-hoc Bonferroni test were used whenever appropriate.

Associations between the categories of body mass index and sociodemographic variables were analyzed using multivariable logistic regression analysis, separately for males and females. The dependent variables formed six different models: each body mass index category vs. normal weight as the referent category; and obesity (including all subjects with body mass index ≥ 30 kg/m^2^) versus normal weight as the referent category. The independent variables were: age, region, type of settlement, educational level, marital status, employment status and wealth index. These were reported with odds ratios and their 95% confidence intervals, along with the probability P. All statistical analyses were performed using the Statistical Package for the Social Sciences (SPSS) version 21.0 software (SPSS Inc., Chicago, IL, USA) and STATA version 11.1 (StataCorp LP, College Station, TX, USA) and the complex sampling design was taken into account. Statistical significance was set at two-sided P < 0.05.

## RESULTS

The study included 12,461 participants of both sexes, with mean age 48.8 ± 17.0 years. Slightly more females than males participated in the study (51.8% versus 48.2%) and the predominant age group among the participants was 20-44 years (42.9%). The highest proportion of the participants was from the Šumadija and Western Serbia region and the majority of the participants lived in urban settlements (60.2%). Most of the participants were married (65.2%), had had secondary education (57.5 %), were employed (37.2%) and belonged to the low wealth index group (41.1%). Out of the whole study population, 36.4% were overweight (BMI 25-29.9 kg/m^2^) and 22.4% were obese (BMI ≥ 30 kg/m^2^). The mean BMI was 26.6 kg/m^2^. Overweight was significantly more frequent among men than among women (Table 1).

**Table 1:** Characteristics of survey participants according to sex and age groups and their differences. Republic of Serbia, 2013

Characteristics	All n = 12,461	Sex		P^‡^	Age groups			P^§^
		Male n = 6,008 (48.2%)	Female n = 6,453 (51.8%)		20-44 n = 5,340 (42.9%)	45-64 n = 4,708 (37.8%)	≥ 65 n = 2,413 (19.3%)	
Age, mean ± SD	48.8 ± 17.0	47.9 ± 16.7	49.6 ± 17.2		32.3 ± 7.1	54.9 ± 5.7	73.4 ± 6.1	
Region, n (%)				0.288				< 0.001
Vojvodina	3,317 (26.6)	1,586 (26.4)	1,731 (26.8)		1,422 (26.6)	1,257 (26.7)	639 (26.5)	
Belgrade	2,904 (23.3)	1,363 (22.7)	1,541 (23.9)		1,322 (24.8)	1,043 (22.2)	539 (22.3)	
Šumadija and Western Serbia	3,419 (27.4)	1,672 (27.8)	1,747 (27.1)		1,466 (27.5)	1,344 (28.5)	609 (25.2)	
Eastern and Southern Serbia	2,821 (22.6)	1,387(23.1)	1,434 (22.2)		1,130 (21.2)	1,065 (22.6)	626 (25.9)	
Settlement, n (%)				< 0.001				< 0.001
Urban	7,498 (60.2)	3,528 (58.7)	3,970 (61.5)		3,352 (62.8)	2,816 (59.8)	1,329 (55.1)	
Rural	4,963 (39.8)	2,480 (41.3)	2,483 (38.5)		1,987 (37.2)	1,892 (40.2)	1,084 (44.9)	
Education level, n (%)				< 0.001				< 0.001
Low	3,070 (24.6)	1,110 (18.5)	1,960 (30.4)		635 (11.9)	1,169 (24.8)	1,265 (52.4)	
Medium	7,161 (57.5)	3,815 (63.5)	3,346 (51.9)		3,628 (67.9)	2,741 (58.2)	794 (32.9)	
High	2,230 (17.9)	1,083 (18.0)	1,147 (17.8)		1,077 (20.2)	798 (17.0)	354 (14.7)	
Marital status, n (%)				< 0.001				< 0.001
Married/living with partner	8,123 (65.1)	4,045 (67.3)	4,078 (63.2)		3,069 (57.5)	3,675 (78.1)	1,379 (57.1)	
Living without partner*	4,338 (34.9)	1,963 (32.7)	2,375 (36.8)		2,270 (42.5)	1,033 (21.9)	1,035 (42.9)	
Employment status, n (%)				< 0.001				< 0.001
Employed	4,640 (37.2)	2,720 (45.3)	1,920 (29.8)		2,780 (52.1)	1,847 (39.2)	14 (0.6)	
Inactive^†^	4,728 (38.0)	1,782 (29.6)	2,946 (45.6)		1,815 (43.7)	1,216 (25.8)	62 (2.6)	
Unemployed	3,093 (24.8)	1,506 (25.1)	1,587 (24.6)		225 (4.2)	1,645 (34.9)	2,337 (96.9)	
Wealth index, n (%)				0.117				< 0.001
Low	5,116 (41.1)	2,523 (42.0)	2,593 (40.2)		1,799 (33.7)	1,997 (42.4)	1,322 (54.8)	
Medium	2,485 (19.9)	1,183 (19.7)	1,302 (20.2)		1,061 (19.9)	996 (21.2)	428 (17.7)	
High	4,860 (39.0)	2,302 (38.3)	2,558 (39.6)		2,480 (46.4)	17,15 (39.5)	664 (27.5)	
BMI, mean ± SD	26.6 ± 5.0	26.9 ± 4.3	26.3 ± 5.6	< 0.001	25.0 ± 4.7	27.8 ± 4.9	27.8 ± 4.9	< 0.001
BMI 25-29.9 kg/m^2^, n (%)	4,541 (36.4)	2,605 (43.3)	1,936 (31.1)		2,384 (32.5)	3,261 (44.5)	1,688 (23.0)	
BMI ≥ 30 kg/m^2^, n (%)	2,793 (22.4)	1,285 (21.6)	1,506 (24.6)		5,340 (42.9)	4,704 (37.8)	2,413 (19.4)	

SD = standard deviation; BMI = body mass index. *Unmarried, divorced or widowed; ^†^Economically inactive (students, disabled people, pensioners and housewives); ^‡^According to chi-square test, t test or Mann-Whitney test, as appropriate; ^§^According to chi-square test, one-way analysis of variance (ANOVA) or Kruskal-Wallis test, as appropriate.

Significant differences were noticed for all variables according to age and gender (with the exception of gender distribution in certain geographical regions). Therefore, further analyses were performed separately for men and women and the prevalences of the BMI categories were adjusted according to age. The prevalences of overweight/obesity according to sociodemographic variables, after adjustment for age, are shown in Table 2 (for men) and Table 3 (for women).

**Table 2:** Prevalence of body mass index (BMI) categories among men, in percentages with 95% confidence intervals (95% CI), adjusted for age, Republic of Serbia, 2013

Characteristics	BMI categories						P^‡^
	Underweight (BMI < 18.50)	Normal weight (BMI = 18.50-24.99)	Overweight (BMI = 25.00-29.99)	Obesity grade I (BMI = 30.00-34.99)	Obesity grade II (BMI = 35.00-39.99)	Obesity grade III (BMI ≥ 40.00)	
BMI, mean ± SE	17.5 ± 0.2	22.7 ± 0.0	27.3 ± 0.0	31.9 ± 0.0	36.7 ± 0.1	42.6 ± 0.2	
Age, mean ± SD	41.3 ± 17.7	44.8 ± 18.3	48.6 ± 15.9	51.6 ± 14.7	52.5 ± 14.4	51.1 ± 12.5	
All	1.1 (0.8-1.3)	34.0 (32.8-35.2)	43.3 (42.1-44.6)	17.3 (16.3-18.2)	3.6 (3.1-4.0)	0.7 (0.5-0.9)	
Age groups							< 0.001
20-44	0.3 (0.0-1.1)	28.3 (24.6-32.0)	48.3 (44.4-52.1)	17.9 (14.9-20.8)	4.3 (2.9-5.8)	0.9 (0.3-1.6)	
45-64	1.1 (0.6-1.6)	31.4 (29.1-33.6)	42.4 (40.0-44.8)	19.9 (18.1-21.7)	4.2 (3.3-5.1)	1.0 (0.6-1.4)	
≥ 65	2.6 (1.4-3.7)	49.9 (44.8-55.1)	35.0 (29.6-40.4)	11.6 (7.5-15.8)	1.0 (0.0-3.0)	0.1 (0.0-0.8)	
Region							0.283
Vojvodina	0.8 (0.3-1.3)	34.4 (32.1-36.7)	44.3 (41.9-46.7)	15.8 (13.9-17.6)	3.9 (3.0-4.8)	0.8 (0.4-1.2)	
Belgrade	0.7 (0.1-1.3)	32.3 (29.7-34.9)	45.1 (42.3-47.8)	18.1 (16.0-17.6)	3.1 (2.1-4.2)	0.7 (0.2-1.1)	
Šumadija and Western Serbia	1.1 (0.6-1.6)	34.1 (31.9-36.3)	42.6 (40.3-44.9)	17.7 (15.9-19.4)	3.7 (2.9-4.6)	0.9 (0.5-1.3)	
Eastern and Southern Serbia	1.6 (1.1-2.2)	34.9 (32.5-37.3)	41.7 (39.2-44.2)	17.8 (15.9-19.7)	3.4 (2.5-4.4)	0.5 (0.1-0.9)	
Settlement							0.049
Urban	0.9 (0.5-1.2)	33.7 (32.1-35.2)	44.7 (43.1-46.4)	16.7 (15.4-17.9)	3.3 (2.6-3.9)	0.8 (0.5-1.1)	
Rural	1.3 (0.9-1.7)	34.5 (32.7-36.3)	41.5 (39.6-43.4)	18.1 (16.7-19.5)	4.0 (3.3-4.7)	0.6 (0.3-1.0)	
Education level							< 0.001
Low	1.8 (1.2-2.4)	40.4 (37.7-43.1)	37.2 (34.4-40.1)	16.0 (13.8-18.2)	3.9 (2.8-4.9)	0.7 (0.2-1.2)	
Medium	1.0 (0.7-1.4)	32.5 (31.0-34.0)	44.2 (42.6-45.8)	17.6 (16.4-18.8)	3.8 (3.2-4.4)	0.8 (0.5-1.1)	
High	0.5 (0.0-1.1)	32.1 (29.3-34.9)	47.0 (44.1-50.0)	17.6 (15.3-19.8)	2.3 (1.2-3.4)	0.5 (0.0-1.0)	
Marital status							< 0.001
Married/living with partner	0.6 (0.3-0.9)	29.7 (28.2-31.1)	45.2 (43.7-46.8)	19.5 (18.4-20.7)	4.1 (3.6-4.7)	0.8 (0.5-1.1)	
Living without partner*	2.1 (1.6-2.6)	43.2 (41.1-45.3)	39.3 (37.0-41.6)	12.5 (10.8-14.2)	2.3 (1.5-3.2)	0.6 (0.2-1.0)	
Employment status							< 0.001
Employed	0.5 (0.1-1.0)	27.2 (25.2-29.1)	48.0 (45.9-50.0)	19.7 (18.1-21.3)	3.8 (3.1-4.5)	0.8 (0.5-1.1)	
Inactive^†^	1.4 (0.7-2.0)	41.4 (38.4-44.3)	39.3 (36.2-42.4)	14.4 (12.1-16.8)	3.9 (2.9-5.0)	0.8 (0.4-1.3)	
Unemployed	1.6 (1.1-2.2)	36.6 (34.2-39.1)	40.5 (37.9-43.1)	16.6 (14.7-18.6)	3.1 (2.4-3.9)	0.6 (0.2-0.9)	
Wealth index							< 0.001
Low	1.7 (1.3-2.1)	37.7 (35.9-39.5)	39.3 (37.4-41.2)	16.7 (15.3-18.1)	7.9 (7.0-8.8)	2.6 (2.0-3.1)	
Medium	0.7 (0.2-1.3)	31.6 (29.0-34.3)	46.1 (43.3-48.9)	16.7 (14.6-18.8)	5.7 (4.5-6.9)	2.1 (1.3-2.8)	
High	0.5 (0.0-0.9)	30.9 (28.9-32.8)	46.7 (44.6-48.8)	18.3 (16.7-19.9)	3.8 (2.8-4.7)	1.8 (1.2-2.4)	

SE = standard error; SD = standard deviation. *Unmarried, divorced or widowed; ^†^Economically inactive (students, disabled persons, pensioners and housewives); ^‡^According to chi-square test or analysis of variance (ANOVA), as appropriate.

**Table 3:** Prevalence of body mass index (BMI) categories among women, in percentages with 95% confidence intervals (95% CI), adjusted for age. Republic of Serbia, 2013

Characteristics	BMI categories						P^‡^
	Underweight (BMI < 18.50)	Normal weight (BMI = 18.50-24.99)	Overweight (BMI = 25.00-29.99)	Obesity grade I (BMI = 30.00-34.99)	Obesity grade II (BMI = 35.00-39.99)	Obesity grade III (BMI ≥ 40.00)	
BMI, mean ± SE	17.7 ± 0.1	22.2 ± 0.0	27.3 ± 0.0	32.0 ± 0.0	36.9 ± 0.1	43.0 ± 0.1	
Age, mean ± SD	37.7 ± 19.2	43.2 ± 16.7	54.2 ± 15.8	57.6 ± 14.0	57.0 ± 14.0	55.8 ± 13.6	
All	3.3 (2.9-3.8)	40.9 (39.8-42.1)	31.1 (30.0-32.2)	16.5 (15.6-17.4)	5.9 (5.4-6.5)	2.2 (1.8-2.5)	
Age groups							< 0.001
20-44	6.6 (5.7-7.6)	61.8 (60.0-63.7)	20.7 (16.1-22.2)	7.1 (6.1-8.0)	2.6 (2.0-3.2)	1.0 (0.6-1.4)	
45-64	1.0 (0.6-1.4)	32.4 (30.5-34.2)	35.7(33.8-37.6)	20.4 (18.8-22.0)	7.3 (6.3-8.4)	2.9 (2.3-3.6)	
≥ 65	2.1 (1.3-2.9)	25.1 (22.8-27.4)	38.2 (35.6-40.7)	23.6 (21.3-25.8)	8.4 (6.9-9.9)	2.4 (1.5-3.2)	
Region							< 0.001
Vojvodina	3.3 (2.4-4.1)	39.9 (37.6-42.1)	31.5 (29.4-33.7)	16.6 (14.9-18.4)	6.3 (5.1-7.4)	2.4 (1.7-3.1)	
Belgrade	3.9 (3.0-4.8)	47.2 (44.9-49.6)	27.7 (25.4-30.1)	14.4 (12.5-16.2)	4.7 (3.4-5.9)	2.1 (1.4-2.9)	
Šumadija and Western Serbia	3.2 (2.5-4.0)	39.9 (37.8-41.9)	31.9 (29.9-34.0)	17.4 (15.8-19.0)	5.6 (4.5-6.6)	2.0 (1.3-2.6)	
Eastern and Southern Serbia	3.1 (2.2-3.9)	37.7 (35.4-39.9)	32.7 (30.5-34.9)	17.2 (15.4-19.0)	7.2 (6.0-8.3)	2.2 (1.5-2.9)	
Settlement							< 0.001
Urban	3.2 (2.7-3.8)	43.3 (41.9-44.8)	30.6 (29.2-32.1)	16.0 (14.8-17.1)	4.9 (4.2-5.6)	1.9 (1.5-2.4)	
Rural	3.5 (2.9-4.2)	37.6 (35.9-39.3)	31.8 (30.1-33.4)	17.2 (15.9-18.6)	7.4 (6.5-8.2)	2.5 (2.0-3.1)	
Education level							< 0.001
Low	4.2 (3.4-5.0)	34.7 (32.6-36.7)	29.9 (27.9-31.9)	19.3 (17.7-20.9)	8.2 (7.2-9.3)	3.7 (3.1-4.4)	
Medium	2.9 (2.3-3.5)	41.5 (39.9-43.1)	32.1 (30.5-33.6)	16.4 (15.1-17.6)	5.5 (4.7-6.3)	1.7 (1.2-2.2)	
High	3.0 (1.9-4.0)	51.9 (49.2-54.7)	30.6 (27.9-33.3)	11.3 (9.2-13.5)	2.6 (1.2-3.9)	0.6 (0.0-1.5)	
Marital status							< 0.001
Married/living with partner	1.9 (1.3-2.4)	37.5 (36.1-38.9)	33.5 (32.2-34.9)	18.1 (17.0-19.2)	6.7 (6.0-7.4)	2.2 (1.8-2.7)	
Living without partner*	5.9 (5.2-6.6)	46.9 (45.0-48.7)	26.9 (25.1-28.7)	13.7 (12.3-15.1)	4.5 (3.6-5.5)	2.1 (1.5-2.7)	
Employment status							< 0.001
Employed	1.3 (0.4-2.2)	49.5 (47.1-51.8)	31.1 (28.9-33.4)	13.3 (11.5-15.1)	3.7 (2.5-4.9)	1.1 (0.4-1.8)	
Inactive^†^	3.9 (3.1-4.7)	36.2 (34.1-38.3)	31.5 (29.5-33.6)	19.0 (17.4-20.6)	6.7 (5.7-7.8)	2.7 (2.0-3.4)	
Unemployed	4.5 (0.5-5.4)	40.1 (34.1-38.3)	30.4 (28.1-32.8)	15.7 (13.8-17.5)	6.9 (5.7-8.1)	2.4 (1.7-3.2	
Wealth index							< 0.001
Low	4.1 (3.5-4.8)	37.6 (35.8-39.3)	30.6 (28.9-32.3)	17.2 (15.9-18.6)	7.9 (7.0-8.8)	2.6 (2.0-3.1)	
Medium	2.5 (1.6-3.5)	39.3 (36.8-41.7)	32.9 (30.4-35.3)	17.6 (15.6-19.5)	5.7 (4.5-6.9)	2.1 (1.3-2.8)	
High	2.9 (2.2-3.6)	45.8 (43.9-47.6)	30.7 (28.9-32.5)	15.1 (13.6-16.5)	3.8 (2.8-4.7)	1.8 (1.2-2.4)	

SE = standard error; SD = standard deviation. *Unmarried, divorced or widowed; ^†^Economically inactive (students, disabled persons, pensioners and housewives).; ^‡^According to chi-square test or analysis of variance (ANOVA), as appropriate.

There were significant differences (P < 0.001) in the distribution of overweight and obesity among men across age groups. Most of the overweight men were in the 20-44 age group and most of the obese men belonged to the 45-64 age group. The prevalences of overweight and obesity differed significantly in relation to all sociodemographic characteristics with the exception of geographical region (Table 2). Most of the overweight men were living in urban settlements, had high educational level, were married, were employed and had a high wealth index. Men with BMI ≥ 30 kg/m^2^ more frequently lived in rural settlements, had secondary education, were married, were employed and had a low wealth index (Table 2).

The prevalences of both overweight and obese women were highest in the age groups ≥ 65 years age group (38.2% and 34.4% respectively, P < 0.001), and overweight and obesity differed significantly according to all sociodemographic characteristics. Most of the overweight and obese women were from Eastern and Southern Serbia (32.7% and 26.6% respectively) and were married (33.5% and 27.0%, respectively). Unlike the men, higher numbers of overweight and obese women lived in rural settlements (31.8% and 27.1% respectively) and were inactive (pensioners or housewives) (31.5% and 28.4% respectively). The largest proportion of the overweight women had received secondary education (32.1%) and were in the medium wealth index group (32.9%). Most of the obese women were of low education level (31.2%), were inactive (28.4%) and had a low wealth index (27.7%) (Table 3).

The associations of BMI categories with sociodemographic variables according to multivariable logistic regression are shown in Tables 4**and**5 for men and women respectively. Among the men, the multivariable logistic regressions showed that the participants who belonged to the 45-64 age group were more obese than those aged 65 years and over, as were also those who lived in rural settlements. The men who were living without a partner and were economically inactive were less frequently obese than were the men who were married and employed, respectively. Grade I obesity was significantly positively related to the richest group according to the wealth index (Table 4). Among the men, the risk of being overweight was higher for those with medium and high education levels, as well as for those with medium and high wealth indexes, while the men living without a partner were less likely to be overweight. Underweight was significantly more frequent among the men who were living without a partner (Table 4).

**Table 4:** Associations of body mass index categories among men with demographic and socioeconomic variables, according to multivariable logistic regression. Republic of Serbia, 2013

Demographic and socioeconomic variables	Odds ratios (95% confidence intervals)*					
	Underweight versus normal	Overweight versus normal	Obesity I versus normal	Obesity II versus normal	Obesity III versus normal	Obesity (all) versus normal
Age groups						
20-44	0.48 (0.10-2.24)	0.82 (0.62-1.10)	0.86 (0.60-1.22)	1.21 (0.65-2.27)	1.19 (0.26-5.46)	0.89 (0.64-1.25)
45-64	0.50 (0.12-2.06)	1.14 (0.89-1.46)	1.55 (1.15-2.09)	2.07 (1.25-3.46)	2.64 (0.71-9.89)	1.63 (1.23-2.16)
> 65	1.00	1.00	1.00	1.00	1.00	1.00
Settlement						
Urban	1.00	1.00	1.00	1.00	1.00	1.00
Rural	0.83 (0.46-1.49)	1.09 (0.94-1.26)	1.24 (1.02-1.50)	1.25 (0.88-1.78)	0.71 (0.34-1.45)	1.20 (1.00-1.44)
Education level						
Low	1.00	1.00	1.00	1.00	1.00	1.00
Medium	0.84 (0.45-1.59)	1.28 (1.08-1.52)	1.16 (0.93-1.45)	1.18 (0.80-1.75)	1.23 (0.55-2.74)	1.16 (0.94-1.42)
High	0.53 (0.18-1.53)	1.39 (1.11-1.74)	1.21 (0.90-1.61)	0.84 (0.47-1.49)	0.77 (0.22-2.76)	1.12 (0.85-1.47)
Marital status						
Married/living with partner	1.00	1.00	1.00	1.00	1.00	1.00
Living without partner^†^	2.42 (1.36-4.33)	0.63 (0.55-0.72)	0.49 (0.41-0.60)	0.44 (0.30-0.64)	0.49 (0.22-1.09)	0.48 (0.41-0.57)
Employment status						
Employed	1.00	1.00	1.00	1.00	1.00	1.00
Inactive^‡^	1.53 (0.84-2.77)	0.74 (0.64-0.86)	0.71 (0.58-0.87)	0.69(0.48-1.03)	1.12 (0.55-2.28)	0.72 (0.60-0.87)
Unemployed	0.52 (0.12-2.17)	0.86 (0.68-1.09)	0.92 (0.69-1.23)	1.19 (0.73-1.93)	0.68 (0.23-2.01)	0.94 (0.72-1.24)
Wealth index						
Low	1.00	1.00	1.00	1.00	1.00	1.00
Medium	0.59 (0.27-1.28)	1.23 (1.04-1.47)	1.07 (0.85-1.35)	1.23 (0.82-1.85)	0.95 (0.42-2.17)	1.10 (0.89-1.36)
High	0.52 (0.24-1.14)	1.29 (1.09-1.53)	1.27 (1.00-1.61)	1.16 (0.74-1.80)	0.68 (0.27-1.68)	1.21 (0.98-1.51)

*Adjusted for region; ^†^Unmarried, divorced or widowed; ^‡^Economically inactive (students, disabled persons, pensioners and housewives). Normal (0) and other body mass index categories (1); 1.00: referent value; the dependent variables formed six different models: each body mass index category vs. normal weight as referent category, and obesity (including all subjects with body mass index ≥ 30 kg/m^2^) versus normal weight as referent category.

**Table 5:** Associations of body mass index categories among women with demographic and socioeconomic variables, according to multivariable logistic regression. Republic of Serbia, 2013

Demographic and socioeconomic variables	Odds ratios (95% confidence intervals)*					
	Underweight versus normal	Overweight versus normal	Obesity I versus normal	Obesity II versus normal	Obesity III versus normal	Obesity (all) versus normal
Age groups						
20-44	2.65 (1.38-5.08)	0.38 (0.29-0.48)	0.32(0.24-0.44)	0.42 (0.26-0.65)	1.06(0.53-2.14)	0.37 (0.29-0.49)
45-64	0.75 (0.40-1.41)	0.91 (0.74-1.12)	0.99 (0.79-1.25)	1.17 (0.84-1.63)	2.38 (1.43-3.97)	1.10 (0.89-1.35)
> 65	1.00	1.00	1.00	1.00	1.00	1.00
Settlement						
Urban	1.00	1.00	1.00	1.00	1.00	1.00
Rural	1.01 (0.71-1.41)	1.01 (0.86-1.18)	0.96 (0.79-1.17)	0.96(0.73-1.28)	0.97 (0.62-1.52)	0.95 (0.80-1.13)
Education level						
Low	1.00	1.00	1.00	1.00	1.00	1.00
Medium	0.78 (0.51-1.18)	0.82 (0.69-0.98)	0.73 (0.59-0.90)	0.73 (0.55-0.98)	0.35 (0.22-0.55)	0.68 (0.57-0.82)
High	0.75 (0.44-1.27)	0.77 (0.62-0.97)	0.48 (0.35-0.65)	0.37 (0.22-0.62)	0.14 (0.05-0.36)	0.41 (0.31-0.54)
Marital status						
Married/living with partner	1.00	1.00	1.00	1.00	1.00	1.00
Living without partner^†^	1.95 (1.45-2.61)	0.62 (0.54-0.71)	0.62 (0.52-0.74)	0.56 (0.42-0.73)	0.85 (0.57-1.27)	0.60 (0.51-0.70)
Employment status						
Employed	1.00	1.00	1.00	1.00	1.00	1.00
Inactive^‡^	1.77 (1.02-3.07)	1.58 (1.31-1.92)	2.01 (0.57-2.56)	2.31 (1.57-3.42)	2.87 (1.52-5.41)	2.13 (1.71-2.65)
Unemployed	2.07 (1.46-2.92)	1.09 (0.92-1.29)	1.23 (0.97-1.55)	1.89 (1.30-2.76)	2.37 (1.28-4.42)	1.43 (1.16-1.76)
Wealth index						
Low	1.00	1.00	1.00	1.00	1.00	1.00
Medium	0.62 (0.40-0.95)	1.13 (0.94-1.36)	1.15 (0.92-1.45)	0.90 (0.65-1.26)	1.30 (0.77-2.19)	1.05 (0.86-1.29)
High	0.66 (0.44-0.98)	1.01 (0.84-1.22)	1.07 (0.85-1.35)	0.61 (0.42-0.87)	1.27 (0.74-2.18)	0.93 (0.75-1.14)

*Adjusted for region; ^†^Unmarried, divorced or widowed; ^‡^Economically inactive (students, disabled persons, pensioners and housewives). Normal (0) and other body mass index categories (1); 1.00: referent value; the dependent variables formed six different models: each body mass index category vs. normal weight as referent category, and obesity (including all subjects with body mass index ≥ 30 kg/m^2^) versus normal weight as referent category.

Among the women, the risk of obesity increased with age, and it was significantly higher among those who were married, had low education level, were economically inactive and were unemployed (Table 5). High wealth index was associated with lower risk of grade II obesity among women (Table 5). Overweight was less frequent among women who were in the youngest age group, who had had secondary and higher education and were living without partner. Underweight was significantly more frequent among young women, women living without a partner, women who were economically inactive or unemployed and those with low wealth index (Table 5).

## DISCUSSION

Our study showed that there were significant socioeconomic inequalities regarding nutritional status. These data, from a nationally representative sample of Serbian adults aged 20 years and over, suggest that low socioeconomic status, as measured according to education, employment and wealth index, was associated with obesity. Moreover, gender differences regarding the association between socioeconomic status and obesity were found.

The primary findings from many studies in middle and low-income countries have shown that there is a strong and direct relationship between socioeconomic status and overweight/obesity, both among men and among women.^9^^,^^10^ This implies that the higher the socioeconomic status is, the more frequent obesity also is. The results from high-income countries have implied an inverse relationship between socioeconomic status and obesity, both among men and among women.^7^^,^^8^ Serbia belongs to the category of upper middle-income countries, with a poverty rate above 10%, and our results show that the prevalence of obesity was highest among those who belonged to the lowest socioeconomic class, among women but not among men.

This relationship between socioeconomic status and obesity, shown through our study, is similar to findings from several other studies in middle-income countries such as Thailand, the Philippines and China.^17^^,^^18^^,^^19^ These studies have suggested that a transition is taking place regarding the relationship between socioeconomic status and obesity, from the pattern of middle-income countries to the pattern of high-income countries, and that this pattern was comparatively faster among women than among men. In Brazil, Monteiro et al. also suggested that obesity was increasing faster among low socioeconomic status groups.^20^ Gender differences regarding the association between socioeconomic status and obesity are complex and may be explained by other factors such as health-related behavior and sociocultural norms.^21^^,^^22^ Social pressure to be slim is stronger among women, particularly in higher socioeconomic status groups.^23^ Among men, overweight and obesity occur more frequently among those who belong to middle and high social classes, according to the wealth index, and among those with middle and higher education levels. The absence of any association between low socioeconomic status and overweight/obesity among men can possibly be explained through the fact that men with low socioeconomic status are more likely to have physically demanding professions.

In our study, a low level of education was associated with higher risk of being overweight and obese, among women, while this was not the case among men. Many studies have found an inverse relationship between educational attainment and obesity, although direct, null and U-shaped associations have also been observed.^24^ The EPIC Panacea study observed that in all the countries involved, body mass index was significantly lower in all higher education categories, compared with the lowest education level. The same study showed that among women, but not among men, the difference between highest and lowest education status was still statistically significant among non-obese subjects.^10^ Sabanayagam et al.^25^ showed in relation to an adult Chinese population that there was an inverse relationship between the level of education and the prevalence of overweigh/obesity among women, such that the highest prevalence of obesity was among the women whose education level was primary school or lower. In the same study, the prevalence of overweight/obesity was lowest among men with post-secondary education.^25^

In our study, both overweight and obese women who were economically inactive and obese women who were unemployed showed positive associations with body mass index. In contrast, there was a negative association with body mass index among men who were economically inactive.

Ball et al. demonstrated associations between employment status and body mass index among women, after controlling for age.^26^ Women who were employed full-time had lower body mass indexes and presented lower risk of overweight than did women who scored lower regarding the employment factor. The relationships observed among these factors were less consistent for men. The relationship between employment and the risk of overweight was the reverse of that among women: men who scored lower in the employment domain were at lower risk of being overweight than were those with higher scores.

There are very few studies in the literature describing the relationship between socioeconomic status and being underweight.^27^^,^^28^ In our study, underweight was more frequent among adults of both sexes who lived in rural settlements, had low education level, were living without a partner, were unemployed and had a low wealth index. Men older than 65 years were more frequently underweight, while among women, the frequency of underweight was highest among those aged 20-44 years.

The main strength of our study is that it used a large representative sample. However, there were several limitations. First, the cross-sectional study design does not allow the causality of the relationship between socioeconomic status and BMI to be considered. Second, the self-reporting of socioeconomic data may be biased and may lead to exposure misclassification. This may attenuate or overestimate relationships between socioeconomic status and obesity. Third, we did not explore mediating factors, such as dietary intake and sedentary behavior.

## CONCLUSION

Our study showed gender-specific associations of socioeconomic status with body mass index among Serbian adults. The results from this study can be generalized for the entire adult population in Serbia, since a nationally representative sample was used. Continuous monitoring of socioeconomic patterns in relation to weight is important, especially for further exploration of the link between education and obesity, since this may lead to development of appropriate education-based policies to counteract recent trends regarding obesity. Further studies are needed to clarify the underlying mechanisms in the relationship between socioeconomic status and obesity.
